# Effects of Continuous Positive Airway Pressure on Body Composition in Individuals with Obstructive Sleep Apnea: A Non-Randomized, Matched Before-After Study

**DOI:** 10.3390/jcm8081195

**Published:** 2019-08-10

**Authors:** Ari Shechter, Michael Airo, Jordan Valentin, Nicholas C. Dugas, Marwah Abdalla, Marie-Pierre St-Onge, Irene K. Louh

**Affiliations:** 1 Center for Behavioral Cardiovascular Health, Columbia University Irving Medical Center, New York, NY 10032, USA; 2 Sleep Center of Excellence, Columbia University Irving Medical Center, New York, NY 10032, USA; 3 Institute of Human Nutrition, Columbia University Irving Medical Center, New York, NY 10032, USA; 4 New York Obesity Nutrition Research Center, Columbia University Irving Medical Center, New York, NY 10032, USA

**Keywords:** obstructive sleep apnea, sleep, obesity, body composition

## Abstract

A reciprocal relationship between obesity and obstructive sleep apnea (OSA) likely exists, wherein obesity contributes to OSA, and OSA-related sleep disturbances promote weight gain. It remains unclear whether continuous positive airway pressure (CPAP) affects body composition. We conducted an open-label, parallel-arm, non-randomized, matched before-after study in individuals with OSA who were starting CPAP use (*n* = 12) and who were not (*n* = 12) to examine the effects of CPAP on total body composition (via air displacement plethysmography) including fat and fat-free mass. CPAP users (*n* = 12) were studied at baseline and after 8 weeks of CPAP use, and 12 age- and sex-matched non-CPAP OSA controls were studied at baseline and after an 8 week period. Statistically significant group x time interactions were seen for body weight, fat-free mass, and fat-mass, such that body weight and fat-free mass were increased, and fat mass decreased, at 8-week follow-up in the CPAP group compared to baseline. Body weight and body composition measures were unchanged in the non-CPAP control group. These findings are consistent with prior studies showing CPAP-induced weight gain, and suggest that weight gain observed following CPAP may be driven primarily by increases in fat-free mass. An increase in lean mass (and decrease in fat mass), despite an overall increase in body weight, can be considered a favorable metabolic outcome in response to CPAP use.

## 1. Introduction

Obstructive sleep apnea (OSA) is associated with increased risk for cardiometabolic morbidities including hypertension, type 2 diabetes, coronary artery disease events and cardiovascular death [1,2,3,4,5,6]. Obesity is a leading risk factor for OSA, and body mass index (BMI), visceral adiposity, and body fat are positively associated with OSA severity [7,8,9,10].

Continuous positive airway pressure (CPAP) is an established treatment for OSA. While the effectiveness of CPAP in preventing cardiovascular events remains controversial [11,12], CPAP use has been shown to improve sleep quality [13], reduce daytime sleepiness [14], increase health-related quality of life [15], and lower blood pressure [16]. CPAP use has also been found to affect some parameters associated with energy balance regulation [17], and may therefore be expected to influence body weight. However, whether, and how CPAP use affects body weight in individuals with OSA is also controversial. Whereas an earlier non-randomized study found that CPAP use is associated with weight loss [18], a meta-analysis of randomized controlled trials (RCTs) reported that CPAP results in a significant increase in body weight [19]. With few exceptions, most studies have focused on body weight, as opposed to other relevant aspects of body composition like fat mass and fat-free/lean mass.

To address these limitations, we conducted a study to examine the effects of CPAP on total body composition, including fat and fat-free mass. Despite recent advances in the field, our initial hypothesis was that CPAP use would be associated with a reduction in body weight and body fat content.

## 2. Methods

This was an open-label, parallel-arm, non-randomized, matched before-after study. One group included CPAP users, in whom measures were taken at baseline, before treatment start, and after 8 weeks of CPAP use. Controls were non-CPAP users, who underwent measures at baseline and 8-week follow-up. Twenty-nine participants were enrolled. One CPAP user dropped out after enrollment but before baseline assessments, 3 CPAP users and one control completed baseline assessment but did not return for 8-week follow-up. Twelve CPAP users and 12 controls completed the study. 

Participants were recruited from the community. Recruitment flyers, which contained a brief summary of the study, general inclusion criteria, and contact information, were placed in local newspapers and were posted around the Columbia University Medical Center campus and online. Inclusion criteria were: recent (within 1 year) diagnosis of at least mild severity OSA (apnea-hypopnea index (AHI) ≥ 5 events/h), BMI ≥ 25 kg/m^2^, and age 18–65 years. Exclusion criteria were: current or prior CPAP use, type 2 diabetes, pregnancy, using anti-psychotic medications, hypnotics, or sleep aids, being a commercial driver or having any recent near-miss or prior car crashes. Body composition was assessed via air displacement plethysmography (BOD POD Body Composition Tracking System, COSMED, Concord, California, USA) [20]. The institutional review board (IRB) of Columbia University Medical Center approved experimental procedures and all participants provided informed written consent (ClinicalTrials.gov Identifier: NCT01944020).

Two-way between-subjects analysis of variance (ANOVA) for repeated measures compared outcomes between groups and across follow-up. Body weight was considered the primary outcome, with fat mass and fat-free mass as secondary outcomes. Since there was a single primary outcome (body weight) and the secondary outcomes (fat and fat-free mass) were considered exploratory and subsidiary, we did not adjust for multiple testing. Statistically significant interactions (*p* < 0.05) were followed up with pairwise comparisons. Data are expressed as mean ± standard deviation (SD), unless otherwise indicated. Analyses were conducted using SPSS, V25.0 (IBM Corp., Armonk, NY, USA).

## 3. Results

CPAP and controls were matched for age, BMI, sex, and baseline AHI (Table 1). A statistically significant group x time interaction was seen for body weight (*p* = 0.04, Table 2): values were higher at follow-up vs. baseline in CPAP (*p* = 0.01), but unchanged in controls (*p* = 0.82). A statistically significant group x time interaction was seen for fat mass % and fat-free mass % (*p*-values = 0.02, Table 2): values were unchanged in controls (*p*-values > 0.05), but fat-free mass % was higher (*p* = 0.04), and fat mass % lower (*p* = 0.04), at follow-up vs. baseline in CPAP (*p* = 0.04). A statistically significant group × time interaction was seen for fat-free mass (in pounds, lbs, *p* = 0.004): values were higher at follow-up vs. baseline in CPAP (*p* = 0.004) but unchanged in controls (*p* = 0.21). For fat mass (lbs), the group × time interaction did not reach statistical significance (*p* = 0.09, Table 2). A lack of statistical power due to a small sample size may contribute to this trend of not reaching statistical significance. For fat mass (lbs), the study had 40% observed power to detect a significant group x time interaction.

## 4. Discussion

We observed that 8 weeks of CPAP was associated with increased body weight and fat-free mass. Similar changes in body composition were not seen in individuals with a recent diagnosis of OSA who were not using CPAP. This is consistent with a meta-analysis of RCTs showing statistically significant, though relatively modest (Hedges’ g = 0.17) post-CPAP increases in body weight [19]. Few studies have examined the effects of CPAP on body composition by looking at changes in fat mass and fat-free/lean mass. Another meta-analysis concluded that CPAP had no effect on visceral fat [21], though others have found post-CPAP increases in lean body mass [22]. Current findings suggest that post-CPAP increases in body weight are driven by increases in fat-free/lean mass (vs. body fat).

The regulation of energy balance and body weight in OSA is complex, and the disorder appears to affect appetite/hunger hormones to promote food intake and to alter energy metabolism [23]. CPAP use may affect energy balance-regulating parameters, e.g., by reducing abnormal leptin and ghrelin levels, or by reducing metabolic rate [17]. While not studied here, increases in growth hormone, which is lipolytic, and insulin-like growth factor-1 (IGF-1), which promotes muscle growth, after CPAP use may also contribute to weight gain and in particular increases in lean/fat-free mass following CPAP use [24].

The sample size used in this study was small, and groups were not randomized, so potential biases may affect outcomes. The non-randomized nature and potential selection bias implies that some factors other than CPAP use per se may have contributed to body composition changes. For example, participants who self-selected to pursue CPAP treatment may have demonstrated improvements in other lifestyle factors not assessed here. There was also some degree of incomplete outcomes data in the study, with three CPAP users and one non-CPAP control completing the baseline assessment but not returning for 8-week follow up. Although groups were ultimately matched for demographics and sample size, given the already modest sample sizes, there may therefore be some degree of attrition bias contributing to the findings as well.

## 5. Conclusions 

The findings of the present study suggest that weight gain observed in individuals with OSA using CPAP may be driven by increases in fat-free mass. However, a definitive conclusion based on the current study is challenging due to the biases resulting from the small sample size and its non-randomized nature. Still, an increase in lean mass (and decrease in fat mass) despite an overall increase in body weight can be considered a favorable metabolic outcome. A more complete understanding of how CPAP affects body composition and energy balance, via larger controlled trials utilizing appropriate assessment methods, will help refine behavioral approaches to treat OSA. For instance, a lifestyle weight loss intervention targeting diet and physical activity was shown to reduce OSA severity [25,26]. Combining these recommendations, particularly a reduced-energy diet, with CPAP, could be used to encourage optimal outcomes for weight management and, potentially, downstream obesity-related cardiometabolic health outcomes.

## Figures and Tables

**Table 1:** Participant demographics at baseline.

	Full Sample (*n* = 24)	CPAP Group (*n* = 12)	No CPAP Group (*n* = 12)	*p*-Value
Age, years	50.4 (10.9)	47.7 (10.9)	53.1 (11.6)	0.25
Female, *n* (%)	10 (42.0%)	5 (42%)	5 (42%)	1.00
BMI, kg/m^2^	35.2 (5.2)	35.6 (4.0)	34.9 (6.5)	0.76
AHI, events/h	30.6 (21.4)	32.6 (21.6)	28.5 (6.5)	0.64

AHI: Apnea-hypopnea index; BMI: Body mass index; CPAP: Continuous positive airway pressure. Data are expressed as mean (SD) or *n* (%). The *p*-value is used for the comparison between CPAP and no CPAP groups.

**Table 2:** Body composition measures between groups and across follow-up.

	CPAP Group (*n* = 12)		No CPAP Group (*n* = 12)		Group (*p*-Value)	Time (*p*-Value)	Group × Time (*p*-Value)
	PRE	POST	PRE	POST			
Body weight, lbs	219.1 (29.5) *	222.3 (30.1) *	223.3 (44.5)	223.2 (46.6)	0.87	0.07	0.04
Fat mass, lbs	87.8 (21.3)	85.7 (19.4)	85.5 (32.8)	87.5 (32.2)	0.98	0.97	0.09
Fat-free mass, lbs	131.3 (23.3) *	136.5 (25.4) *	137.8 (30.0)	135.7 (28.9)	0.80	0.19	0.004
Fat mass, %	40.0 (7.6) *	38.7 (7.3) *	37.7 (11.2)	38.6 (10.4)	0.75	0.61	0.02
Fat-free mass, %	60.0 (7.6) *	61.4 (7.3) *	62.3 (11.2)	61.4 (10.4)	0.75	0.61	0.02

CPAP: Continuous positive airway pressure; PRE: baseline; POST: 8-week follow up. Data are expressed as mean (SD). *p*-values are from two-way between subjects ANOVA for repeated measures (factors: group × time), and are shown for the main effects of Group and Time, and for the Group × Time interaction. Bold denotes *p* < 0.05. * indicates statistically significant pairwise comparisons (CPAP group PRE vs. CPAP group POST; *p* < 0.05).
